# Synthesis and Structural Characterization of Silver Nanoparticles Stabilized with 3-Mercapto-1-Propansulfonate and 1-Thioglucose Mixed Thiols for Antibacterial Applications

**DOI:** 10.3390/ma9121028

**Published:** 2016-12-20

**Authors:** Francesco Porcaro, Laura Carlini, Andrea Ugolini, Daniela Visaggio, Paolo Visca, Ilaria Fratoddi, Iole Venditti, Carlo Meneghini, Laura Simonelli, Carlo Marini, Wojciech Olszewski, Nitya Ramanan, Igor Luisetto, Chiara Battocchio

**Affiliations:** 1 Department of Sciences, Roma Tre University, Via della Vasca Navale 79, 00146 Rome, Italy; laura.carlini@uniroma3.it (L.C.); and.ugolini2@gmail.com (A.U.); daniela.visaggio@uniroma3.it (D.V.); paolo.visca@uniroma3.it (P.V.); carlo.meneghini@uniroma3.it (C.M.); igor.luisetto@uniroma3.it (I.L.); chiara.battocchio@uniroma3.it (C.B.); 2 Department of Chemistry, Sapienza University, P.le A. Moro 5, 00085 Rome, Italy; ilaria.fratoddi@uniroma1.it (I.F.); iole.venditti@uniroma1.it (I.V.); 3 Alba Synchrotron Facility, Carrer de la Llum, 2-26, Cerdanyola del Vallès, 08290 Barcelona, Spain; lsimonelli@cells.es (L.S.); cmarini@cells.es (C.M.); wolszewski@cells.es (W.O.); nramanan@cells.es (N.R.); 4 Faculty of Physics, University of Bialystok, 1L K. Ciolkowskiego street, 15-245 Bialystok, Poland

**Keywords:** silver nanoparticles, antibacterial activity, hydrophilic ligands

## Abstract

The synthesis, characterization and assessment of the antibacterial properties of hydrophilic silver nanoparticles (AgNPs) were investigated with the aim to probe their suitability for innovative applications in the field of nanobiotechnology. First, silver nanoparticles were synthetized and functionalized with two capping agents, namely 3-mercapto-1-propansulfonate (3MPS) and 1-β-thio-d-glucose (TG). The investigation of the structural and electronic properties of the nano-systems was carried out by means of X-ray Photoelectron Spectroscopy (XPS) and X-ray Absorption Spectroscopy (XAS). XPS data provided information about the system stability and the interactions between the metallic surface and the organic ligands. In addition, XPS data allowed us to achieve a deep understanding of the influence of the thiols stoichiometric ratio on the electronic properties and stability of AgNPs. In order to shed light on the structural and electronic local properties at Ag atoms sites, XAS at Ag K-Edge was successfully applied; furthermore, the combination of Dynamic Light Scattering (DLS) and XAS results allowed determining AgNPs sizes, ranging between 3 and 13 nm. Finally, preliminary studies on the antibacterial properties of AgNPs showed promising results on four of six multidrug-resistant bacteria belonging to the ESKAPE group (*Enterococcus faecium*, *Staphylococcus aureus*, *Klebsiella pneumoniae*, *Acinetobacter baumannii*, *Pseudomonas aeruginosa*, and *Enterobacter* sp.).

## 1. Introduction

Nowadays, the human ability to treat serious infections is threatened by the incessant evolution of antimicrobial resistance. In particular, bacterial pathogens belonging to the ESKAPE group (*Enterococcus faecium*, *Staphylococcus aureus*, *Klebsiella pneumoniae*, *Acinetobacter baumannii*, *Pseudomonas aeruginosa*, and *Enterobacter* sp.) “escape” (are resistant to) the antibacterial activity of currently available antibiotics. Infections spread by these species represent a major public health threat since they cannot be cured by conventional therapies [1]. ESKAPE bacteria are particularly dreaded in the hospital environment because of their responsibility in a variety of nosocomial infections. In addition, they show a dramatic tendency to pan-resistance, i.e., the resistance to all available antibiotics [1]. This results in higher morbidity and mortality, with huge social and economic costs [2]. Within this context, recent efforts to address this challenge lie in the exploration of antimicrobial nanomaterials, to which microbial pathogens may not be able to develop resistance [3]. Among others, AgNPs were considered, in recent years, particularly attractive for the production of a new class of antimicrobials, opening up a completely new way to combat a wide range of bacterial pathogens [4]. The mechanistic aspects responsible for the AgNPs bactericide effects are far from being fully elucidated. Despite that, it is generally accepted that the Lewis acid cations Ag+ amount delivered inside the bacterial cell are mainly responsible for the therapeutic effect [5]. Bearing this in mind, two important key points have guided the development of the most recent synthetic approaches: (1) reducing the NPs size in order to maximize the surface atoms number; and (2) functionalizing NPs surface with proper ligands in order to promote the uptake in cells [6]. Noteworthy, the chemical synthetic process based on the reduction in solution of silver precursor salts offers an higher yield, easier production and lower costs compared to other methods [7]. However, regardless of whatever synthetic strategy is selected, the great therapeutic potential of AgNPs also relies on the amazing chemical versatility achievable by surface modifications: a huge variety of ligands (antibiotics, peptides, and nucleic acids) can be conjugated to the metal nanoparticles through non-covalent and covalent interactions [8]. Despite that, the capping of metallic clusters deeply affects the NPs chemo-physical stability. Indeed, the selection of the proper ligand can lead to the formation of different shells reducing the overall stability of the system to the external environment [9]. Moreover, as already demonstrated by our group with gold nanoparticles, simple monosaccharides functionalization can impact on the NPs fate and lead to increased uptake by cells [10].

In the framework of this research topic emerges a double fundamental role played by nanostructure surface: its antibacterial efficacy and its influence in the NPs outcome. Consequently, a deeper investigation of the nanomaterial structure is mandatory in order to understand and further improve new therapeutic applications.

In the present work, the system is composed by AgNP-s functionalized with two capping agents, 3-mercapto-1-propansulfonate (3MPS) and 1-β-thio-d-glucose (TG), selected on purpose for, respectively, stabilizing the particles and exploiting the bacterial chemical affinity for simple sugars. X-ray Absorption Spectroscopy (XAS) can shed light on the local properties at Ag atoms sites composing the nanostructure. In addition, X-ray Photoelectron Spectroscopy (XPS) can be successfully applied to understand the behavior of organometallic interfaces and to investigate the chemical composition of such systems. Furthermore, a preliminary determination of IC_50_ and IC_90_ was carried out with the aim to test the concrete biological effect of this new kind of nanostructure on Bacterial pathogens belonging to the ESKAPE group.

## 2. Results and Discussion

### 2.1. Ag-3MPS-TG Nanoparticles Synthesis and Purification

**Figure 1:**
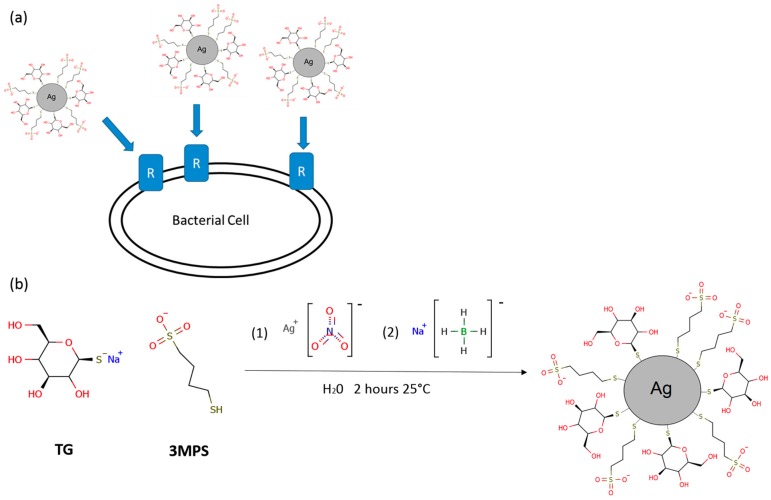
(**a**) Targeting of bacterial cells by means of nanoparticles surface functionalization. Glucose functionalization can induce a selective interaction between nanoparticles and Glucose receptors expressed by bacterial cells promoting the uptake; (**b**) General reaction scheme of AgNPs-3MPS-TGs nanoparticles synthesis. The reaction involves the Ag reduction by means of NaBH_4_. Briefly, to a 3MPS and TG solution, Ag solution is added (1). Subsequently, the NaBH_4_ is added (2) in order to induce the metal reduction leading to the nanostructure formation.

Several syntheses of silver nanoparticles were performed adding different quantities of TG molecules keeping constant silver nitrate and 3MPS amount. 3MPS gives a hydrophilic character to the nanoparticles while TG is expected to improve bacterial cells targeting, exploiting a selective binding to bacterial glucose receptor as shown in Figure 1a. The AgNPs synthesis consisted on typical wet reduction of silver nitrate to metallic silver by means of sodium borohydride [11,12]. Attempts to produce AgNPs stabilized with TG alone gave rise to aggregated structures not suitable as colloidal suspensions. The choice of introducing 3MPS was made considering its stabilization efficiency, and the hydrophilic and negative surface charge character of the already studied AuNPs-3MPS [10,13]. Consequently, on the basis of our previous studies, the AgNPs synthesis was carried out by varying the Ag/3MPS/TG ratio. We explored a variety of molar ratios, 1/4/0, 1/4/0.1, 1/4/0.5 and 1/4/1 (named AgNPs-3MPS, AgNPs-3MPS-TG1, AgNPs-3MPS-TG2, and AgNPs-3MPS-TG3, respectively), in order to explore the effect on size and properties of the nanoparticles and the binding efficiency at the AgNPs surface. Unfortunately, the molar ratio 1/4/1 (sample AgNPs-3MPS-TG3) gave a totally aggregated product, probably due to the high amount of TG which is responsible for an increased interaction between particles through hydrogen bonding networks present in carbohydrates [14]. For this reason, we mainly focused our attention to 1/4/0.1 and 1/4/0.5 molar ratios. The reaction scheme is shown in Figure 1b. Purification steps were performed by centrifugation and dialysis, as reported in the experimental. AgNPs-3MPS-TGs were characterized by means of UV–visible spectroscopy, DLS, XAS, XPS and Transmission Electron Microscopy (TEM). In Table S1 of Supplementary Materials, the experimental data collected on AgNPs-3MPS, AgNPs-3MPS-TG1 and AgNPs-3MPS-TG2 are reported as a comparison. The UV–visible spectra reported show the typical Surface Plasmon Resonance (SPR) band in the range 400–500 nm. Consequently, the lack of SPR band in the AgNPs-TG spectrum has been considered as a marker for the loss of NPs stability due to the 3MPS absence.

DLS measurements confirm the nanometric dimensions of the AgNPs-3MPS-TG and AgNPs-3MPS-TG2 samples in liquid phase (aqueous solution), showing <2R_H_> = 3 ± 1 nm and <2R_H_> = 6 ± 2 nm, respectively (data reported in Supplementary Materials Figures S1–S4). Moreover, the Z-potential studies evidence the negative charged surface and the stability of AgNPs in water suspension, showing Z-potential = −40 ± 5 mV and Z-potential = −38 ± 4 mV for AgNPs-3MPS-TG1 and AgNPs-3MPS-TG2, respectively [15]. A sum up of AgNPs main physiochemical proprieties is reported in Table 1.

### 2.2. Structural Characterization

#### 2.2.1. X-ray Absorption Spectroscopy

**Figure 2:**
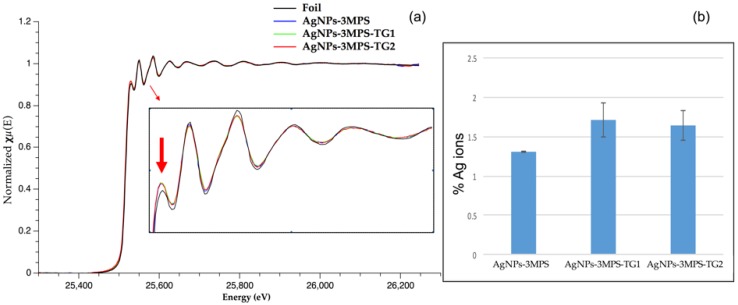
(**a**) Normalized Spectra of NPs and Foil samples and Ag^+^ determination. Inset: Zoom on XANES region shows the raising of white line intensity (arrow) and decreasing of fine structure oscillations in NPs with respect to the bulk reference; (**b**) Results of Ag^+^ sample quantification by means of subtraction method. The methodology is further described in Materials and Methods Section.

The X-ray Absorption Near Edge Structure (XANES) investigation can provide qualitative information concerning the nanoparticles structural arrangement and their electronic proprieties [16]. Indeed, from the comparison of normalized nanoparticles spectra in the XANES region (Figure 2) two important features were observed. First, the white line intensity (Figure 2a onset) showed an increased intensity respect to the foil. Such intensity enhancement can be explained in terms of photoelectron transition to increased density of new Ag empty states. Consequently, the formation of Ag-S bonds is in agreement with above picture implying the Ag p-orbitals emptying due electrons migration to more electronegative atoms like sulfur. A tentative linear combination by several Ag-S reference compounds has been performed leading to lack of consistent results.

However, by means of Ag foil normalized spectrum subtraction, an amount of 1.2%–1.7% Ag^+^ was finally determined in each sample. (Figure 2b inset).

Second, it is noteworthy that the early oscillations above the edge following to the white line were dumped with respect to the foil. This behavior was consistent with an average coordination number (CN) reduction or an increase in the system disorder. The decrease of the metal–metal CN is a well-documented phenomenon of metal NPs, relying on the increase of surface atoms which have fewer neighboring atoms, hence causing the overall CN to decrease [17]. However, in order to assess quantitatively a possible CN reduction, the EXAFS region was investigated in more detail.

**Figure 3:**
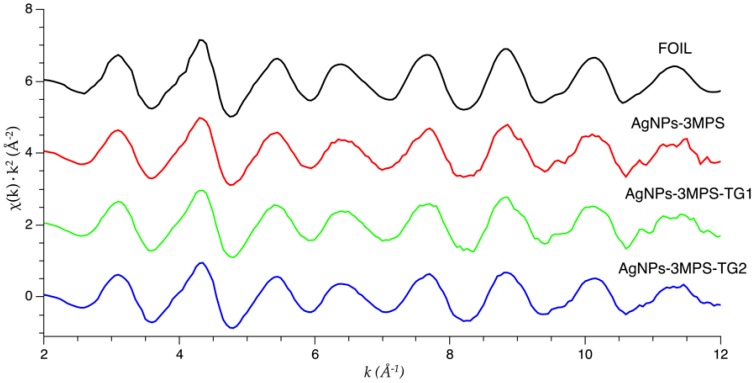
*k*^2^ weighted Ag K-edge EXAFS spectra of the Ag-reference metal foil (top curve) and the data measured on AgNPs (vertically shifted for clarity).

Raw EXAFS data of AgNPs-3MPS, AgNPs-3MPS-TG1, and AgNPs-3MPS-TG2 samples were converted into *k*-space. Figure 3 shows that the oscillatory patterns of the *k*-space spectra closely resembled the bulk foil *k*-space, which suggest that the NPs are metallic.

**Figure 4:**
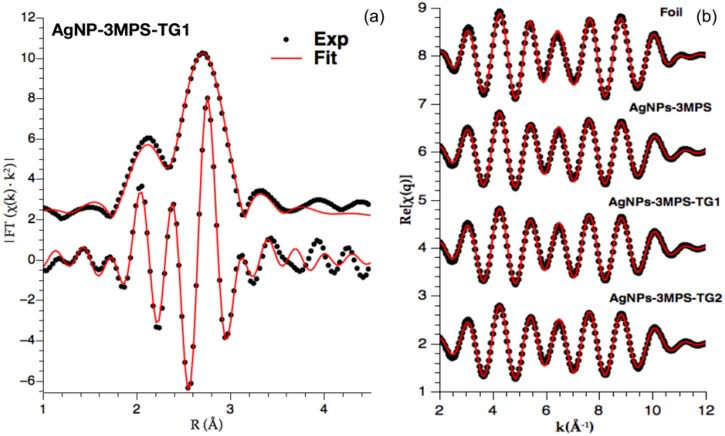
Graphical results of EXAFS analysis: (**a**) Example of EXAFS best fit analysis on Ag-3MPS-TG2 sample: Ag K-edge *k*^2^ weighted Fourier transform modulus (top curves) and imaginary part (bottom curves): experimental (points) and best fit (red full lines) shifted for clarity. The fit takes into account only Ag–Ag single scattering signal path present in first shell; (**b**) Fourier filtered experimental (points) and best fit (ful lines) for reference Ag foil and NPs, vertically shifted for clarity.

In order to obtain quantitative structural information about bonding and structure of AgNPs, the *k*^2^ weighted R-space spectra were refined focusing on the first shell fit. The resulting overall refinements and goodness of the fit for each sample (expressed by the R-factors) are reported in Table 2. The obtained values were all below 0.02 denoting acceptable refinements [18]. As an example, the Ag K-edge AgNPs-3MPS-TG1 spectrum, its related imaginary component, and their fits are shown in Figure 4a in R-Space. Moreover, Fourier filtered experimental and best fit for reference Ag foil and AgNPs were reported in Figure 4b. 

The metallic character of the samples is demonstrated by the Ag–Ag bond lengths shown in Table 2. All the NPs exhibit Ag–Ag distances of around 2.85 Å, which is analogous to the bulk (2.86 Å from experimental R-space refinement), and consistent with a slight lattice contraction due to nanostructuring expected for NPs of this size [19]. Table 1 also shows that the Ag–Ag CN decreases from around 12 (typical for Face Cubic Centered Ag) to around 11 in all the nanoparticles samples. The metal–metal CN decrease confirmed the qualitative clues offered from XANES analysis. Moreover, we signal the raising of disorder factors (σ^2^) associated to the decreasing coordination numbers. Owing to the known positive correlation between CN and σ^2^, this finding is a further validation of reliable CN reduction in NPs.

CN values can be exploited in order to estimate NPs diameter [20,21,22]. A simple model [21] is based on two assumptions: first, the particle shape is spherical; and, second, atoms at the nanoparticle surface only have half of inner atoms CN. Consequently, the average CN is calculated by the formula:

(1) $$\langle N_{0}\rangle \;=N_{x_{in}}+\frac{N_{x_{out}}}{2}$$

where $N_{x_{in}}$ and $N_{x_{out}}$ represent, respectively, the fraction of inner and surface atoms. Applying further geometrical considerations explained in Ref. [21], the final formula was obtained, which allows calculating the particle radius:

(2) $$R_{p}=\frac{3R_{s_{i}}\;N}{2\Delta N}$$

where $R_{p}$ is the NP radius. $R_{s_{i}},N,\;\Delta N$ values represent, respectively, the *i*-th-shell radius, the theoretical *i*-shell CN value, and the difference between theoretical *i*-shell CN value and the refined one. Using the refined values of the first shell and solving the equation, the three different particles diameters were calculated and reported in Table 2. The sizes obtained by means of XAS are consistent with TEM measurements, which showed an average size ranging between 15 and 20 nm (Supplementary Materials Figure S6) for all the samples. Both TEM and XAS techniques are carried out in solid-state phase, and then can be affected by aggregation phenomena not observed in aqueous solution (see DLS data).

In order to further investigate the thiol molecules influence on NPs bulk structure, an attempt to fit the sulfur component from Ag_2_S structure was also performed. However, the best fit does not improve and the Ag-S contribution remains weak. Therefore, the fraction of Ag_2_S phase can be considered negligibly weak in the samples. The small fraction of Ag^+^, responsible for white line raising in the XANES (Figure 2), should be due to the Ag-S bonds at the NP surfaces but their fraction is too weak to be reliably included in the EXAFS fit. We reserved the possibility of further investigating this issue by means of future S K-edge analyses. However, since sulfur should be present mainly on nanoparticles surface, we considered deep investigation by means of XPS mandatory.

#### 2.2.2. X-ray Photoelectron Spectroscopy

The XPS technique is an extremely powerful tool to investigate the chemical and electronic structure at the molecule–metal interface and the ligands stability in capped nanoparticles samples [10]. Since XPS provides complementary information to XAS, being able to probe the surface properties, X-ray photoemission measurements were carried out on all the here proposed AgNPs-3MPS-TGs nanoparticles; for comparison, the model system AgNPs-3MPS was also investigated. XPS data (BE, FWHM, atomic ratios and assignments) collected at S2p, Ag3d and O1s core levels, which are the most indicative for the analysis, are reported in Table 2 (S2p and Ag3d), Table 3 (comparison between S2p data analysis results) and Table 4 (comparison between O1s data analysis results).

Looking at the S2p spectra, two spin–orbit doublets were distinguished. The signal having the main S2p_3/2_ component at about 161.4 eV BE is attributed to sulfur atoms chemically bonded to metal atoms at the NP surface, as expected from the literature [23]. The pair of components at higher binding energy (S2p_3/2_ component ~168–169 eV BE) is related to the sulfonates groups arising by the 3MPS ligand (SO^−^Na^+^ end group) [24]. A signal related to physisorbed thiols, usually found around 163.5 eV [25], is not observable in these samples.

**Figure 5:**
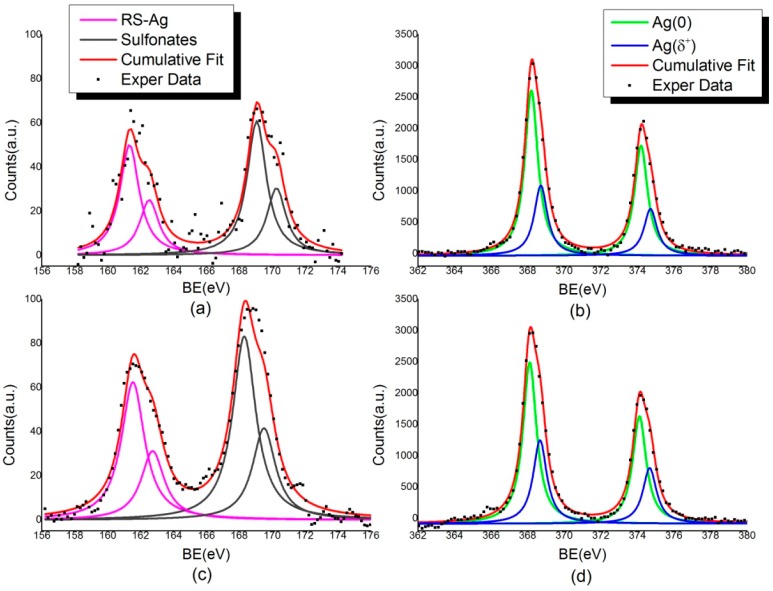
XPS measurements on AgNPs S2p and Ag3d signals: (**a**) S2p and (**b**) Ag3d spectra for AgNPs-3MPS sample; and (**c**) S2p and (**d**) Ag3d spectra for AgNPs-3MPS-TG1 sample.

Ag3d core level spectra confirm the effectiveness of the bond between TG and 3MPS thiol end groups and silver clusters. As shown in Figure 5, a pronounced shoulder in the pure metal Ag3d peak can be observed, and attributed to the silver atoms at the NP surface that are covalently bonded with thiols (nearly 32 atomic percent, as reported in the Supplementary Materials Table S1). The Ag3d_5/2_ component at about 368.2 eV corresponds to unperturbed metallic silver, as expected for atoms in NPs core. The lower intensity peak contributions at higher BE are assigned to positively charged Ag atoms at the NPs surface, chemically bonded to the sulfur atoms of thiol end groups. The two Ag3d spin–orbit pairs are separated of about 0.5–0.6 eV.

With the aim of probing the TG grafting on silver clusters, surface O1s core level spectra were also collected and analyzed. As reported in Table 3, the “–OH– like” O1s component signal intensity increases when TG ligands are inserted in the composite system. All analysis details concerning O1s component signal are reported in Supplementary Materials Table S2.

In order to confirm this trend, the sample with increased TG amount, i.e., AgNPs-3MPS-TG2 (Ag/TG/3MPS = 1/4/0.5), was also analyzed. As shown in Table 3, the trend evidencing the increase of OH-like oxygen atoms amount (component at about 533.5 eV BE, see Table 3) with respect to oxygen atoms of SO^−^ groups and carbonyl groups of surface contaminants (∼532.1 eV BE) going from AgNPs-3MPS (Ag/3MPS = 1/4) to AgNPs-3MPS-TG1 (Ag/3MPS/TG = 1/4/0.1) to AgNPs-3MPS-TG2 (Ag/3MPS/TG = 1/4/0.5) is fully confirmed. The atomic percent of –(OH)– like O1s signal is 4.71 in AgNPs-3MPS, 12.85 in AgNPs-3MPS-TG1 and 15.84 in AgNPs-3MPS-TG2. 

**Figure 6:**
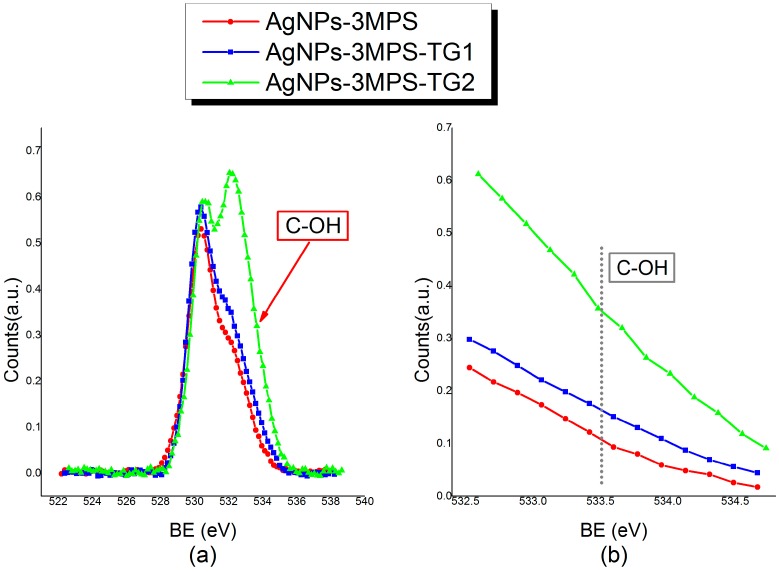
XPS measurements on AgNPs 1Os signals: (**a**) Comparison between O1s spectra for AgNPs-3MPS, AgNPs-3MPS-TG1 and AgNPs-3MPS-TG2 samples. The peak at lower BE (~530 eV) refers to the TiO_2_ signal of the sample support, and it has been chosen as reference component to normalize spectra intensities; (**b**) Detail of the 532.6–534 eV BE region, evidencing the C-OH component intensity trend.

The increasing of the ”–OH– like” O1s component amount following the TG stoichiometry can also be clearly observed in O1s spectra reported in Figure 6.

In conclusion, the collected data fully confirmed the chemical structure proposed at the bottom of Figure 1 for the Ag-3MPS-TG1 sample, consistent with literature reports on analogous materials [10,26]. Moreover, a clear suggestion on synthetic procedure efficacy for surface glucose grafting was obtained.

### 2.3. Antibacterial Activity

The antimicrobial activity of the AgNP preparations AgNPs-3MPS, AgNPs-3MPS-TG1 and AgNPs-3MPS-TG2 was assessed on both Gram-positive and Gram-negative bacteria, including the reference *Escherichia coli* strain ATCC47076 and bacterial strains belonging to the ESKAPE group, namely *S. aureus* ATCC25923, *K. pneumoniae* ATCC27736, *A. baumannii* ATCC19606^T^, *P. aeruginosa* ATCC15692 and *E. faecalis* ATCC29212.

AgNPs concentrations causing 90% reduction of the bacterial growth after 16-h incubation at 37 °C (IC_90_) could be determined for *E. coli* ATCC47076, *P. aeruginosa* ATCC15692 and *E. faecalis* ATCC29212, in a concentration range 31–122 µg/mL (Table 5). Among the three preparations tested, AgNPs-3MPS-TG1 exerted an overall stronger antibacterial effect compared with AgNPs-3MPS and AgNPs-3MPS-TG2. Indeed, for *E. coli* ATCC47076 and *P. aeruginosa* ATCC15692, the IC_90_ values of AgNPs-3MPS-TG1 were less than half of those determined for AgNPs-3MPS and AgNPs-3MPS-TG2 (Table 5). Notably, AgNPs-3MPS-TG1 was also the only AgNP preparation that inhibited the growth of *E. faecalis* ATCC29212 (IC_90_ 59 ± 2.3 µg/mL) and reduced *K. pneumoniae* ATCC27736 growth by 50% (IC_50_; Table 5).

Although, to a lesser extent, AgNPs-3MPS-TG1 and AgNPs-3MPS-TG2 also reduced the growth of *S. aureus* ATCC25923, only IC_50_ values of 59 ± 2.4 and 62 ± 3.3 µg/mL could be determined, respectively. None of the AgNP preparations tested were inhibitory for *A. baumannii* ATCC19606^T^ (IC_50_ > 128 µg/mL; Table 5).

The above results show a promising activity of AgNPs against some bacterial species belonging to high antibiotic resistant ESKAPE group. Interestingly, a possible correlation between NPs activity and glucose functionalization can be envisaged, though this point deserves more in depth investigation.

## 3. Materials and Methods

### 3.1. Chemicals and Materials

Silver Nitrate (AgNO_3_, Sigma-Aldrich, St. Louis, MI, USA, ≥99.9%), sodium borohydride (NaBH_4_, Sigma-Aldrich, 99%), sodium 3-mercapto-1-propanesulfonate (3MPS, C_3_H_7_S_2_O_3_Na, Sigma-Aldrich, 98%), and 1-Thio-β-d-glucose sodium salt (TG, Sigma-Aldrich, 99%) were used. Deionized water was obtained from Zeener Power I Scholar-UV (electrical resistivity 18.2 MΩ) instrument (Human Corporation, Songpa-gu, Seul, Korea). Nitrogen flux was flown through the reaction solvents for the deoxygenation of the reaction mixtures.

### 3.2. Synthesis of AgNPs

The synthesis of the AgNPs was performed through reduction in aqueous phase of AgNO_3_ with NaBH_4_ in the presence of 3MPS and/or TG as capping agents, according to literature reports [10]. The reactions were carried out at room temperature for 2 h.

#### 3.2.1. Synthesis of AgNPs-3MPS

The synthesis of Ag-3MPS nanoparticles was conducted according to literature report, by using an Ag/thiol molar ratio 1/4 [27]. Main characterizations of the products are herein reported Ag-3MPS: UV (λ_max_ (nm), H_2_O): 432 nm; DLS (<2R_H_> (nm), H_2_O): 10 ± 2; Z potential (mV): −33 ± 2.

#### 3.2.2. Synthesis of AgNPs-3MPS-TGs

The TG molar ratios were varied keeping constant amounts of silver salt and the 3MPS: Ag/3MPS/TG = 1/4/0 (AgNPs-3MPS); Ag/3MPS/TG = 1/4/0.1 (AgNPs-3MPS-TG1) Ag/3MPS/TG = 1/4/0.5 (AgNPs-3MPS-TG2). The ratios selected have been chosen in order to avoid the aggregation phenomena occurring in the AgNPs-TG samples; a molar ratio of Ag/3MPS/TG = 1/4/1 gave a totally aggregated product after the purification steps. The procedure for the synthesis of AgNPs-3MPS-TG1 is reported below, as an example. AgNO_3_ (100 mg, 5886 × 10^−4^ mol) was dissolved in 5 mL of deionized H_2_O and subsequently a solution of sodium 3-mercapto-1-propanesulfonate (41,963 mg, 2354 × 10^−3^ mol) and 1-Thio-β-d-glucose sodium salt (1284 mg, 5886 × 10^−5^ mol) in 10 mL of H_2_O was added drop wise into the same reaction pot, under vigorous stirring. The initially clear solution, turned yellow after the addition of the first drops, and a grainy white precipitate is formed; it returns in solution with the addition of all the 10 mL of the thiol solution. After 5 min a nitrogen flux was flown for 15 min through the reaction solvents for the deoxygenation of the reaction ambient. After that, through a syringe, NaBH_4_ (22,263 mg, 5886 × 10^−3^ mol) dissolved in 5 mL of H_2_O was quickly added at the solution. After this step the solution turned deep brown. The reaction was kept at room temperature under stirring for 2 h. The crude reaction product was subjected to one centrifugation cycle at 5000 rpm; to obtain a purified material, the sample was resuspended in deionized water and put into a 14 kDa MWCO dialysis tubing cellulose membrane (Sigma-Aldrich) for 30 days changing the washing waters (deionized) two times per day. The obtained product then was fully characterized. Main characterizations of the products are herein reported. AgNPs-3MPS-TG1: UV (λ_max_ (nm), H_2_O): 434nm; DLS (<2R_H_> (nm), H_2_O): 3 ± 1; Z potential (mV): −40 ± 5; Ag-3MPS-TG2: UV (λ_max_ (nm), H_2_O): 416nm; DLS (<2R_H_> (nm), H_2_O): 6 ± 2; Z potential (mV): −38 ± 4; UV-visible spectra and DLS data are reported in Supplementary Materials Figures S1–S5.

### 3.3. Spectroscopic Methods

#### 3.3.1. UV–Visible Spectroscopy and Dynamic Light Scattering

UV–visible Absorption spectra of AgNPs dispersed in deionized water were measured in 1.00 cm optical path quartz cells by using a Cary 100 Varian. Dynamic light scattering (DLS) measurements, were carried out on the nanoparticle aqueous suspensions (0.50–0.20 mg/mL), using a Brookhaven instrument (Brookhaven, NY, USA) equipped with a 10 mW HeNe laser at a 632.8 nm wavelength at a temperature of 25.0 ± 0.2 °C. ζ potential was measured using the laser Doppler velocimetry technique (Malvern Nano ZS90 instrument, Malvern Instruments Ltd., Worchestershire, UK) and particle velocity was expressed per unit field strength as the electrophoretic mobility u. The ζ potential was calculated using the Henry equation as reported in our previous [28,29].

#### 3.3.2. Transmission Electron Microscopy

Morphological analysis of AgNPs was carried out by means of a Philips CM 120 Analytical Transmission Electron Microscope (FEI, Hillsboro, OR, USA) equipped with Lanthanum Hexaboride (LaB6) gun, energy dispersive X-ray spectrometry (EDS) nanoprobe and double-tilt low-background holder. Images were acquired using a 12-bit SIS Megaview III camera with 1392 × 1040-pixel resolution (EMSIS GmbH, Muenster, Germany). Analyses were performed using an acceleration voltage of 100 kV. Sampling was performed by dipping for 1 s a copper grid (3 mm diameter) with a support film of formvar (polyvinyl formal)/carbon coat inside the solution. The grid was then dried for about 5 min by means of infrared heat lamp and then it was readily introduced into the microscope chamber.

The mean particles diameter $\bar{d}\;(\mathrm{nm})$ and the surface-weighted average diameter ${\bar{d}}_{av}\;(\mathrm{nm})$ were calculated from the number of particle $N_{d_{i}}$ with a diameter d_i_ according to Equations (3) and (4), respectively

(3) $$\bar{d}\;(\mathrm{nm})=\sum_{n}^{i=1}\frac{N_{d_{i}}\cdot d_{i}}{N_{total}}$$

(4) $${\bar{d}}_{av}\;(\mathrm{nm})=\frac{\sum_{n}^{i=1}N_{d_{i}}\cdot d_{i}^{3}}{\sum_{n}^{i=1}N_{d_{i}}\cdot d_{i}^{2}}$$

#### 3.3.3. X-ray Absorption Spectroscopy

EXAFS spectra were collected at the Ag K-edge (25,514 eV) at CLAESS, the X-ray absorption and emission spectroscopy beamline at ALBA synchrotron facility (CELLS, Barcelona, Spain). The synchrotron radiation emitted by a multipole wiggler is vertically collimated by the vertical collimating mirror, than monochromatized by a liquid nitrogen cooled double crystals Si311 monochromator, and finally focused down to sample up to 200 × 200 µm^2^ by toroidal focusing mirror. The spectra were collected in transmission mode by using proper filled ionization chambers as detectors. Liquid solutions sample were disposed in a static liquid cell, which length has been optimized to guarantee the correct absorption signal.

Data analysis (normalization and EXAFS modeling) has been performed using the Demeter package (Version 0.9.25, copyright © 2009–2016 Bruce Ravel) [30].

All spectra have been normalized by subtracting the linear contribution of the pre-edge and dividing by the linear fitting of the post-edge region. To quantify the amount of Ag^+^, we study the absolute difference between normalized subtracted spectra. Specifically, each sample spectrum has been first normalized, then it has been subtracted by the foil normalized spectrum. The absolute difference between the spectra has been then integrated in the range 25,215–26,250 eV. The resulting values are expressed as Ag^+^ percentage.

To get further insight about the local geometry of the nanoparticles, we then focus on the EXAFS part of the spectra. Standard procedure based on the cubic spline fit to the pre-edge subtracted absorption spectra was used to extract the EXAFS signal to determine local structural parameters.

The EXAFS structural refinement we carried out using the standard formula [31]:

(5) $$\chi_{th}\left(k\right)=S_{0}^{2}\underset{i}{\sum }\left[\frac{N_{i}A_{i}\left(k\right)}{kR_{i}^{2}}\right]\cdot \sin[(2kR_{i}+\;\phi_{i}\left(k\right)]e^{\left(-2\sigma_{i}^{2}k^{2}\right)}e^{\left[\frac{-2R_{i}}{\lambda_{i}\left(k\right)}\right]}$$

The theoretical curve *x_th_*(*k*) is modeled as a sum of contributions representing the *i*-th neighbor shells around the absorber, each one being assumed having Gaussian distribution with average distance *R_i_*, variance $\sigma_{i}^{2}$ and multiplicity *N_i_*. The $A_{i}\left(k\right)$ and $\phi_{i}\left(k\right)$ are the amplitude and phase function describing the process of absorption and scattering of the photoelectron, *λ_i_*(*k*) is the photoelectron mean free path, $S_{0}^{2}$ takes into account for many-body losses. Theoretical amplitude, phase and backscattering functions were generated on the basis of crystallographic Ag [32] and structures using FEFF program [33]. The model curves were fitted to experimental data (*k* range from 1.2 to 12 Å^−1^), taking into account the main single scattering contributions in between 1.2 and 3.2 Å, and least square refinement obtained using Demeter [30] program. Experimental data and best fit in k-space and moduli of Fourier transforms (|FT|) are shown in Figure 4a for AgNPs-3MPS-TG1 nanoparticles.

#### 3.3.4. X-ray Photoemission Spectroscopy

XPS measurements were performed on an instrument of our own design and construction, consisting of a preparation and an analysis UHV chamber, equipped with a 150 mm mean radius hemispherical electron analyzer with a four-element lens system with a 16-channel detector giving a total instrumental resolution of 1.0 eV as measured at the Ag3d_5/2_ core level. The analysis chamber is equipped with a six degrees of freedom manipulator and a 150 mm mean radius hemispherical electron analyzer with five-lens output system combined with a 16-channel detector. MgKα non-monochromatized X-ray radiation (hν = 1253.6 eV) was used for acquiring C1s, O1s, S2p and Ag3d signals of all samples. The energies were referenced to the metal core level of the samples and C1s signal of the aromatic carbon atoms was always found at about 285.50 eV BE (binding energy). Atomic ratios were calculated from peak intensities by using calculated λ factors [34]. Curve-fitting analysis of all spectra was performed using Lorentzian profiles as fitting functions with the same FWHM for both spin–orbit components, after subtraction of a Shirley-type background [35]. The core level binding energy (BE) and full width at half-maxima (FWHM) were analyzed with particular attention to the metal and sulfur spin–orbit components. The S2p_3/2, 1/2_ doublet was fitted by using a spin–orbit splitting of 1.2 eV and a branching ratio (S2p_3/2_/S2p_1/2_) of 2. For the Ag3d_5/2, 3/2_ doublet, a splitting of 6 eV and a branch ratio Ag3d_5/2_/Ag3d_3/2_ of 3/2 were used. All samples were deposited onto Si(111) substrates by following a drop-casting procedure.

### 3.4. Microbiology

The strains used in this study are *E. coli* ATCC47076 (alternative designation MG1655, [36]), *S. aureus* ATCC25923, *K. pneumoniae* ATCC27736, *A. baumannii* ATCC19606^T^, *P. aeruginosa* ATCC15692 and *E. faecalis* ATCC29212. Strains were obtained from the American Type Culture Collection (Gaithersburg, MD, USA). Susceptibility testing to AgNPs was performed according to the broth microdilution method in Mueller Hinton broth II (MHBII) [37]. Briefly, nanoparticles were prepared in aqueous solution containing 5 mg/mL of bovine serum albumin (BSA) at the final concentration 512 µg/mL.

BSA was added to the AgNPs suspensions in order to prevent their precipitation in MHBII, and AgNPs were serially diluted in MHBII to achieve the final concentration range 0.25–128 µg/mL. Bacteria were grown in MHBII for 16 h, then diluted in saline and inoculated into AgNPs-supplemented MHBII to a final concentration of ca. 5 × 10^5^ colony forming units (CFU)/mL. Bacterial growth was monitored spectrophotometrically (OD_600_) using a Wallac 1420 Victor3V multilabel plate reader (Perkin Elmer) for 16 h at 37 °C without shaking. The IC_50_ and IC_90_, i.e., the concentrations of AgNPs that inhibited bacterial growth by 50% and 90%, respectively, were calculated using the GraphPad Prism software (version 5.0; GraphPad Software, San Diego, CA, USA) and expressed as mean value of three replicates ± the standard deviation.

## 4. Conclusions

Hydrophilic AgNPs stabilized with TG and 3MPS mixed thiols were synthesized and extensively structurally characterized. The observed CN decrease combined with a slight Ag–Ag bond contraction proved by means XAS spectroscopy confirmed the fulfillment of silver nanocluster formation. By means of DLS and XAS characterization, the NPs size was assessed to vary in the range 3–13 nm. Moreover, the XPS analysis confirmed the theoretical synthetic trend concerning the efficacy of surface glucose grafting. Among the three NPs tested, AgNPs-3MPS-TG1 exerted an overall stronger antibacterial effect compared with AgNPs-3MPS and AgNPs-3MPS-TG2. Indeed, for *E. coli* ATCC47076 and *P. aeruginosa* ATCC15692 the effective antibacterial concentrations of AgNPs-3MPS-TG1 were the less than half of those determined for AgNPs-3MPS and AgNPs-3MPS-TG2. Notably, AgNPs-3MPS-TG1 was also the only AgNPs preparation that inhibited the growth of *E. faecalis* ATCC29212 and reduced *K. pneumoniae* ATCC27736 growth by 50%. In conclusion, we developed new silver nanoparticles stable in water solution, extremely suitable for potential use as antibacterial agents against four out of six highly antibiotic resistant bacterial strains.

## Figures and Tables

**Table 1:** Details of AgNPs.

Sample Name	Molar Ratio			DLS
	Ag	3MPS	TG	Size (nm); Z Potential (mV)
AgNPs-3MPS	1	4	/	10 ± 2; −33 ± 2
AgNPs-3MPS-TG1	1	4	0.1	3 ± 1; −40 ± 5
AgNPs-3MPS-TG2	1	4	0.5	6 ± 2; −38 ± 4

**Table 2:** Ag K-edge R-space refinement results for AgNPs-3MPS, AgNPs-3MPS-TG1, AgNPs-3MPS-TG2 NPs.

Sample	Shell	Bond	CN	R (Å)	σ^2^ (Å^2^)	∆E_0_ (eV)	R-Factor	Diameter (nm)
Ag-Foil	I	Ag–Ag	12 *	2.86 (1)	0.0092 (4)	3.1 (1)	0.009	∞
AgNPs-3MPS	I	Ag–Ag	11.2 (4)	2.85 (1)	0.010 (1)	2.8 (1)	0.010	13 (1)
AgNPs-3MPS-TG1	I	Ag–Ag	11.1 (3)	2.85 (1)	0.010 (1)	2.8 (1)	0.013	11 (1)
AgNPs-3MPS-TG2	I	Ag–Ag	10.8 (4)	2.85 (1)	0.010 (1)	3.0 (1)	0.013	9 (1)

* Indicates parameters kept fixed during the refinements.

**Table 3:** S2p_3/2_ binding energy values and relative atomic ratios of AgNPs-3MPS and AgNPs-3MPS-TG1 samples.

**RS-Ag**	**BE (eV)**	**N_i_/N_tot_**
AgNPs-3MPS	161.33	0.8
AgNPs-3MPS-TG1	161.53	0.7
**Sulfonates**	**BE (eV)**	**N_i_/N_tot_**
AgNPs-3MPS	169.06	1
AgNPs-3MPS-TG1	168.31	1

**Table 4:** O1s binding energy, relative atomic ratio and atomic percent for AgNPs-3MPS and AgNPs-3MPS TG-samples.

**O=C, –SO_3_–**	**BE (eV)**	**N_i_/N_tot_**	**Atomic (%)**
AgNPs-3MPS	532.25	0.46	30.17
AgNPs-3MPS-TG1	532.03	0.56	31.37
AgNPs-3MPS-TG2	532.29	1.09	43.89
**C–OH**	**BE (eV)**	**N_i_/N_tot_**	**Atomic (%)**
AgNPs-3MPS	533.6	0.07	4.71
AgNPs-3MPS-TG1	533.5	0.23	12.85
AgNPs-3MPS-TG2	533.73	0.39	15.84

**Table 5:** Inhibitory concentrations (IC_50_ and IC_90_; μg/mL) of AgNPs-3MPS, AgNPs-3MPS-TG1 and AgNPs-3MPS-TG2 against reference bacterial strains. Experiments were performed in triplicate and mean IC vales are reported ± the standard deviation.

Strain	IC_50_			IC_90_		
	AgNPs-3MPS	AgNPs-3MPS-TG1	AgNPs-3MPS-TG2	AgNPs-3MPS	AgNPs-3MPS-TG1	AgNPs-3MPS-TG2
*E. coli* ATCC47076	55 ± 2.2	27 ± 1.5	33 ± 2.3	122 ± 2.9	56 ± 3.2	119 ± 2.4
*S. aureus* ATCC25923	>128	59 ± 2.4	62 ± 3.3	>128	>128	>128
*K. pneumoniae* ATCC27736	>128	122 ± 2.6	>128	>128	>128	>128
*A. baumannii* ATCC19606^T^	>128	>128	>128	>128	>128	>128
*P. aeruginosa* ATCC15692	98 ± 3.6	25 ± 3.2	88 ±2.6	119 ± 3.1	31 ± 2.5	120 ± 2.7
*E. faecalis* ATCC29212	126 ± 1.2	39 ± 2.4	28 ± 2.2	>128	59 ± 2.3	>128

## Supplementary Materials

The following are available online at www.mdpi.com/1996-1944/9/12/1028/s1. Figure S1: Dynamic Light Scattering of AgNPs-3MPS-TG1, Figure S2: Z potential of AgNPs-3MPS-TG1, Figure S3: Uv-Vis Normalized spetra of AgNPs samples, Figure S4: Dynamic Light Scattering of AgNPs-3MPS-TG2, Figure S5: Z potential of AgNPs-3MPS-TG2, Figure S6: Trasmission Electron Microscopy (TEM) Images. TEM pictures and average size corresponding to all three samples: AgNPs-3MPS: 19 ± 3 (Image a–d); AgNPs-3MPS-TG1: 15 ± 2 (Image b–e); AgNPs-3MPS-TG2: 13 ± 1(Image c–f), Table S1: XPS data collected at S2p and Ag3d core levels on samples AgNPs-3MPS and AgNPs-TG-3MPS, Table S2: XPS data of O1s signal.

## References

[1] Pendleton J.N., Gorman S.P., Gilmore B.F. (2013). Clinical relevance of the ESKAPE pathogens. Expert Rev. Anti-Infect. Ther..

[2] Boucher H.W., Talbot G.H., Bradley J.S., Edwards J.E., Gilbert D., Rice L.B., Scheld M., Spellberg B., Bartlett J. (2009). Bad bugs, no drugs: No ESKAPE! An update from the Infectious Diseases Society of America. Clin. Infect. Dis..

[3] Huh A.J., Kwon Y.J. (2011). “Nanoantibiotics”: A new paradigm for treating infectious diseases using nanomaterials in the antibiotics resistant era. J. Control. Release.

[4] Franci G., Falanga A., Galdiero S., Palomba L., Rai M., Morelli G., Galdiero M. (2015). Silver nanoparticles as potential antibacterial agents. Molecules.

[5] Durán N., Durán M., Bispo de Jesus M., Seabra A.B., Fávaro W.J., Nakazato G. (2016). Silver nanoparticles: A new view on mechanistic aspects on antimicrobial activity. Nanomed. Nanotechnol. Biol. Med..

[6] Singh R., Shedbalkar U.U., Wadhwani S.A., Chopade B.A. (2015). Bacteriagenic silver nanoparticles: Synthesis, mechanism, and applications. Appl. Microbiol. Biotechnol..

[7] Zhang X.F., Liu Z.G., Shen W., Gurunathan S. (2016). Silver Nanoparticles: Synthesis, Characterization, Properties, Applications, and Therapeutic Approaches. Int. J. Mol. Sci..

[8] Rai M., Kon K., Ingle A., Duran N., Galdiero S., Galdiero M. (2014). Broad-spectrum bioactivities of silver nanoparticles: The emerging trends and future prospects. Appl. Microbiol. Biotechnol..

[9] Jiménez-Lamana J., Slaveykova V.I. (2016). Silver nanoparticle behaviour in lake water depends on their surface coating. Sci. Total Environ..

[10] Porcaro F., Battocchio C., Antoccia A., Fratoddi I., Venditti I., Fracassi A., Luisetto I., Russo M.V., Polzonetti G. (2016). Synthesis of functionalized gold nanoparticles capped with 3-mercapto-1-propansulfonate and 1-thioglucose mixed thiols and “in vitro” bioresponse. Colloids Surf. B Biointerfaces.

[11] Brust M., Walker M., Bethell D., Schiffrin D.J., Whyman R. (1994). Synthesis of thiol-derivatised gold nanoparticles in a two-phase Liquid–Liquid system. Chem. Commun..

[12] Zhao P., Li N., Astruc D. (2013). State of the art in gold nanoparticle synthesis. Coord. Chem. Rev..

[13] Cametti C., Fratoddi I., Venditti I., Russo M.V. (2011). Dielectric relaxations of ionic thiol-coated noble metal nanoparticles in aqueous solutions: Electrical characterization of the interface. Langmuir.

[14] Katti K.K., Kattumuri V., Bhaskaran S., Katti K.V., Kannan R. (2009). Facile and general Method for synthesis of sugar coated gold nanoparticles. Int. J. Green Nanotechnol. Biomed..

[15] Fratoddi I., Venditti I., Battocchio C., Polzonetti G., Cametti C., Russo M.V. (2011). Core shell hybrids based on noble metal nanoparticles and conjugated polymers: Synthesis and characterization. Nanoscale Res. Lett..

[16] Mobilio S., Boscherini F., Meneghini C. (2014). Synchrotron Radiation Basics, Methods and Applications.

[17] Liu F., Wechsler D., Zhang P. (2008). Alloy-structure-dependent electronic behavior and surface properties of Au–Pd nanoparticles. Chem. Phys. Lett..

[18] Koningsberger D.C., Mojet B.L., Dorssen G.E., Van Ramaker D.E. (2000). XAFS spectroscopy; fundamental principles and data analysis. Top. Catal..

[19] Padmos J.D., Zhang P. (2012). Surface structure of organosulfur stabilized silver nanoparticles studied with X-ray absorption spectroscopy. J. Phys. Chem. C.

[20] Calvin S., Miller M.M., Goswami R., Cheng S.F., Mulvaney S.P., Whitman L.J., Harris V.G. (2003). Determination of crystallite size in a magnetic nanocomposite using extended X-ray absorption fine structure. J. Appl. Phys..

[21] Battocchio C., Meneghini C., Fratoddi I., Venditti I., Russo M.V., Aquilanti G., Maurizio C., Bondino F., Matassa R., Rossi M. (2012). Silver nanoparticles stabilized with thiols: A close look at the local chemistry and chemical structure. J. Phys. Chem. C.

[22] Jentys A. (1999). Estimation of mean size and shape of small metal particles by EXAFS. Phys. Chem. Chem. Phys..

[23] Zhang S., Leem G., Lee T.R. (2009). Monolayer-protected gold nanoparticles prepared using long-chain alkanethioacetates. Langmuir.

[24] (2012). NIST X-ray Photoelectron Spectroscopy Database.

[25] Battocchio C., Porcaro F., Mukherjee S., Magnano E., Nappini S., Fratoddi I., Quintiliani M., Russo M.V., Polzonetti G. (2014). Gold nanoparticles stabilized with aromatic thiols: Interaction at the molecule–metal interface and ligand arrangement in the molecular shell investigated by SR-XPS and NEXAFS. J. Phys. Chem. C.

[26] Vitale F., Vitaliano R., Battocchio C., Fratoddi I., Giannini C., Piscopiello E., Guagliardi A., Cervellino A., Polzonetti G., Russo M.V. (2008). Synthesis and microstructural investigations of organometallic Pd(II) thiol-gold nanoparticles hybrids. Nanoscale Res. Lett..

[27] Venditti I., Fontana L., Fratoddi I., Battocchio C., Cametti C., Sennato S., Mura F., Sciubba F., Delfini M., Russo M.V. (2014). Direct interaction of hydrophilic gold nanoparticles with dexamethasone drug: Loading and release study. J. Colloid Interface Sci..

[28] Venditti I., Fratoddi I., Palazzesi C., Prosposito P., Casalboni M., Cametti C., Battocchio C., Polzonetti G., Russo M.V. (2010). Self-assembled nanoparticles of functional copolymers for photonic applications. J. Colloid Interface Sci..

[29] Quintiliani M., Bassetti M., Pasquini C., Battocchio C., Rossi M., Mura F., Matassa R., Fontana L., Russo M.V., Fratoddi I. (2014). Network assembly of gold nanoparticles linked through fluorenyl dithiol bridges. J. Mater. Chem. C.

[30] Ravel B., Newville M. (2005). ATHENA, ARTEMIS, HEPHAESTUS: Data analysis for X-ray absorption spectroscopy using IFEFFIT. J. Synchrotron Radiat..

[31] Bunker G. (2010). Introduction to XAFS: A Practical Guide to X-ray Absorption Fine Structure Spectroscopy.

[32] Spreadborough J., Christian J.W. (1959). High-temperature X-ray diffractometer. J. Sci. Instrum..

[33] Zabinsky S.I., Rehr J.J., Ankudinov A., Albers R.C., Eller M.J. (1995). Multiple-scattering calculations of X-ray-absorption spectra. Phys. Rev. B.

[34] Swift P., Shuttleworth D., Seah M.P., Briggs D., Seah M.P. (1983). Quantification of AES and XPS. Practical Surface Analysis by Auger and X-ray Photoelectron Spectroscopy.

[35] Shirley D.A. (1972). High-resolution X-ray photoemission spectrum of the valence bands of gold. Phys. Rev. B.

[36] Soupene E., van Heeswijk W.C., Plumbridge J., Stewart V., Bertenthal D., Lee H., Prasad G., Paliy O., Charernnoppakul P., Kustu S. (2003). Physiological studies of Escherichia coli strain MG1655: Growth defects and apparent cross-regulation of gene expression. J. Bacteriol..

[37] Wiegand I., Hilpert K., Hancock R.E. (2008). Agar and broth dilution methods to determine the minimal inhibitory concentration (MIC) of antimicrobial substances. Nat. Protoc..

