# Supplementary material for: Synthesis and Structural Characterization of Silver Nanoparticles Stabilized with 3-Mercapto-1-Propansulfonate and 1-Thioglucose Mixed Thiols for Antibacterial Applications
Source: Materials (Basel). 2016 Dec 20;9(12):1028. doi: 10.3390/ma9121028 (PMC5456982; doi:10.3390/ma9121028)
Supplement: Supplementary file 1 [file materials-09-01028-s001.pdf]

Supplementary Materials: Synthesis and Structural Characterization of Silver Nanoparticles Stabilized with 3-Mercapto-1-Propansulfonate and 1-Thioglucose Mixed Thiols for Antibacterial Applications

Francesco Porcaro, Laura Carlini, Andrea Ugolini, Daniela Visaggio, Paolo Visca, Ilaria Fratoddi, Iole Venditti, Carlo Meneghini, Laura Simonelli, Carlo Marini, Wojciech Olszewski, Nitya Ramanan, Igor Luisetto and Chiara Battocchio

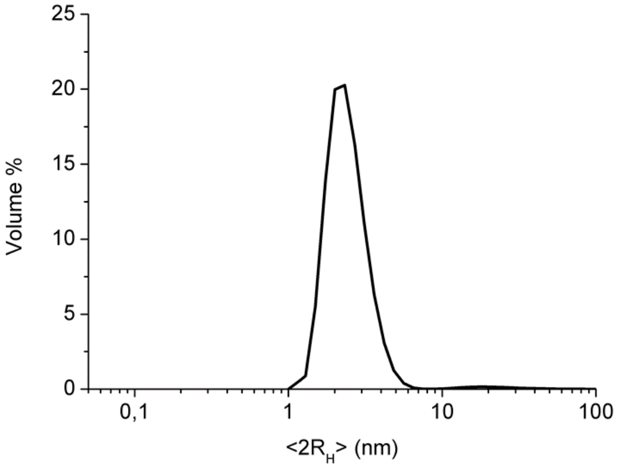

Figure S1. Dynamic Light Scattering of AgNPs-3MPS-TG1.

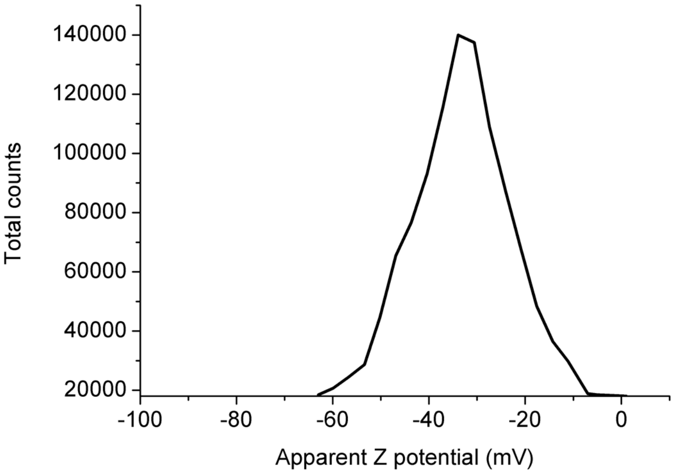

Figure S2. Z potential of AgNPs-3MPS-TG1.

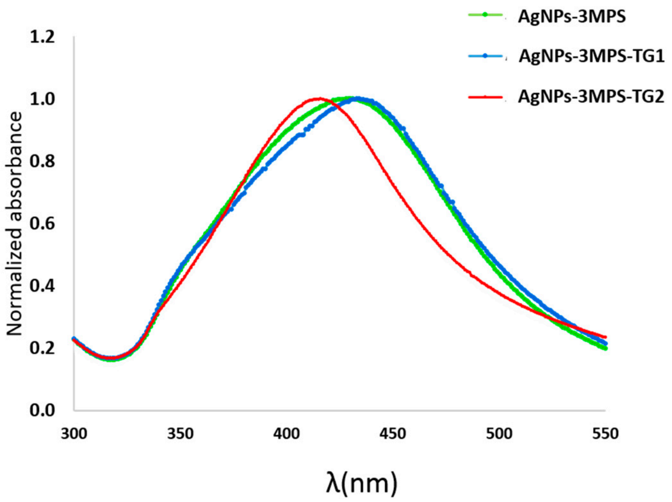

Figure S3. Uv-Vis Normalized spetra of AgNPs samples.

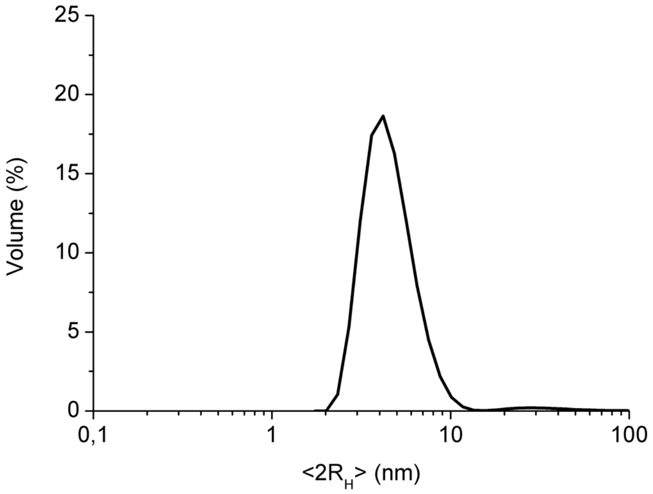

Figure S4. Dynamic Light Scattering of AgNPs-3MPS-TG2.

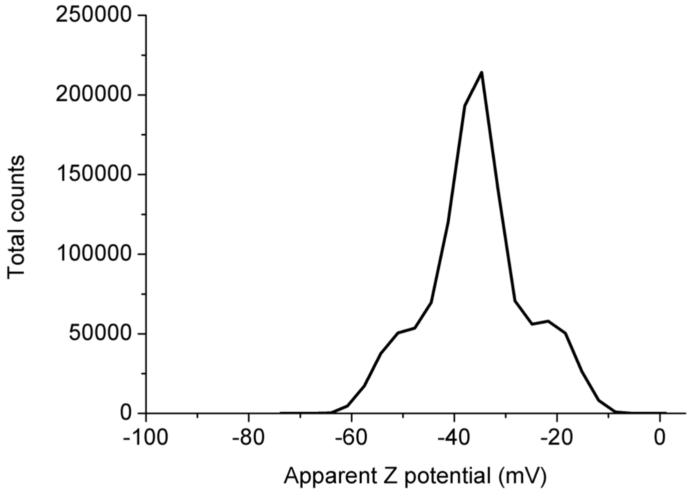

Figure S5. Z potential of AgNPs-3MPS-TG2.

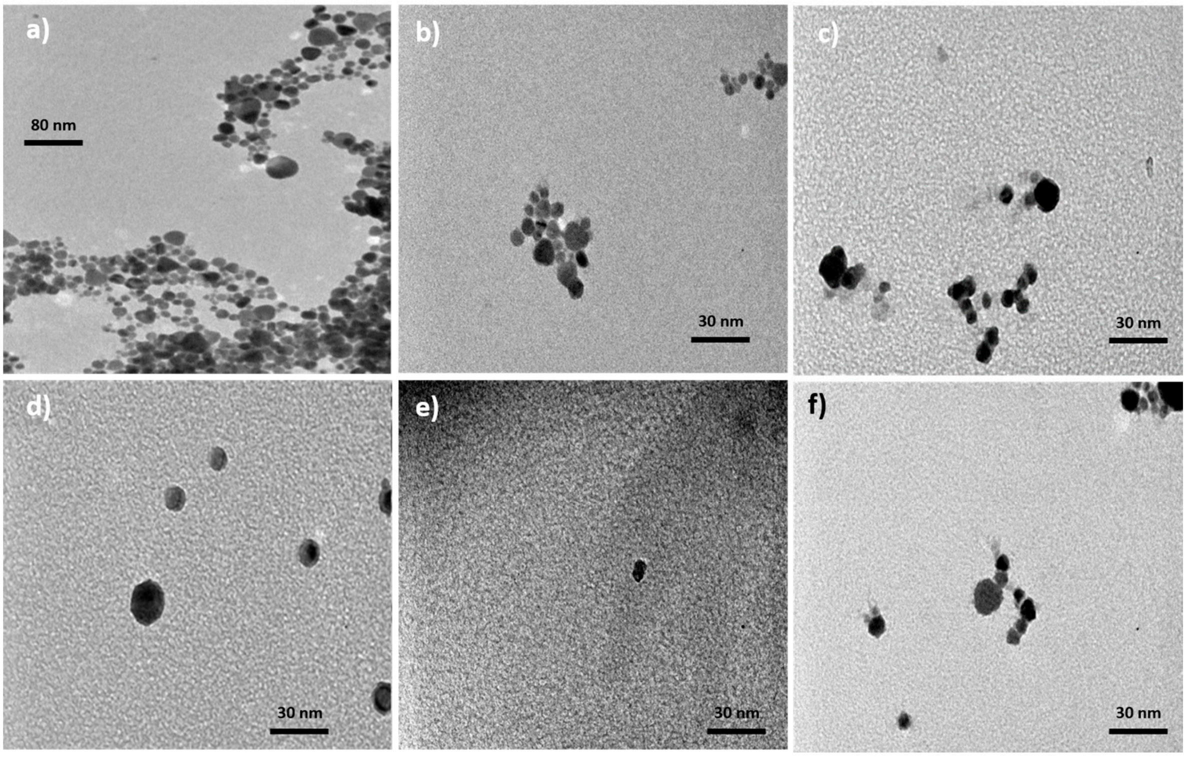

Figure S6. Trasmission Electron Microscopy (TEM) Images. TEM pictures and average size corresponding to all three samples: AgNPs-3MPS: 19 ± 3 (Image **a**–**d**); AgNPs-3MPS-TG1: 15 ± 2 (Image **b**–**e**); AgNPs-3MPS-TG2: 13 ± 1(Image **c**–**f**).

Table S1. XPS data collected at S2p and Ag3d core levels on samples AgNPs-3MPS and AgNPs-TG-3MPS.

Sample	Signal *	BE (eV)	FWHM	Atomic (%)	N_i/N_{tot}	Assignments
AgNPs-3MPS	$S2p_{3/2}$					
	1	161.33	1.41	45.2	0.8	RS-Ag
	3	169.06	″	54.8	1	$SO^{-}Na^{+}$
	$Ag3d_{5/2}$					
	1	368.2	0.87	70.2	1	Ag(0)
	2	368.72	″	29.8	0.42	$Ag(\delta^{+})$
AgNPs-3MPS-TG1	$S2p_{3/2}$					
	1	161.53	1.7	42.4	0.7	RS-Ag
	3	168.31	″	57.6	1	$SO^{-}Na^{+}$
	$Ag3d_{5/2}$					
	1	368.11	0.91	65.9	1	Ag(0)
	2	368.67	″	34.1	0.52	$Ag(\delta^{+})$

* A spin-orbit splitting of 1.2 eV and a branching ratio ($S2p_{3/2}/S2p_{1/2}$) of 2 were used for the sulfur doublets, while a branching ratio ($Ag3d_{5/2}/Ag3d_{3/2}$) of 1.5 and a spin-orbit splitting of 6 eV were used for silver.

Table S2: XPS data of O1s signal.

Sample	O1s	BE(eV)	FWHM	Assignment
AgNPs-3MPS	1	530.35	1.88	TiO_2
	2	532.25	″	O=C, $–SO_3–$
	3	533.6	″	C–OH
AgNPs-3MPS-TG1	1	530.32	1.7	TiO_2
	2	532.03	″	O=C, $–SO_3–$
	3	533.5	″	C–OH
AgNPs-3MPS-TG2	1	530.5	1.72	TiO_2
	2	532.29	″	O=C, $–SO_3–$
	3	533.73	″	C–OH
